# Risk Factors and Environmental Preventive Actions for Aspergillosis in Patients with Hematological Malignancies

**DOI:** 10.3390/clinpract14010022

**Published:** 2024-02-05

**Authors:** Daniel Raposo Puglia, José Ángel Raposo Puglia, Emilio García-Cabrera, Fátima Morales, Juan Carlos Camacho-Vega, Ángel Vilches-Arenas

**Affiliations:** 1 Department of General and Digestive Surgery, Hospital Universitario Jerez de la Frontera, Ronda de Circunvalación s/n, 11407 Jerez de la Frontera, Spain; danielrpuglia@gmail.com; 2 Department of Hematology, Hospital Universitario Puerta del Mar, Ana de Viya, 21, 11009 Cádiz, Spain; josea.raposo.sspa@juntadeandalucia.es; 3 Preventive Medicine and Public Health Department, Faculty of Medicine, University of Seville, Av. Sanchez Pizjuan s/n, 41009 Seville, Spain; fmmarin@us.es (F.M.); ava@us.es (Á.V.-A.); 4 Department of Building Constructions II, Higher Technical School of Building Engineering, University of Seville, Avda. de la Reina Mercedes, 4A, 41012 Seville, Spain; jccamacho@us.es; 5 Occupational Risk Prevention Unit, Virgen Macarena Hospital, Avda. Dr. Fedriani 3, 41009 Seville, Spain; 6 Department of Preventive Medicine, Virgen Macarena Hospital, Avda. Dr. Fedriani 3, 41009 Seville, Spain

**Keywords:** aspergillosis, risk factors, neutropenia, primary prevention, environmental control

## Abstract

(1) Background: *Aspergillus* spp. is a widely distributed filamentous fungus in the environment due to its high sporulation capacity. Currently, invasive aspergillosis (IA) is the most common invasive fungal infection in patients with hematologic malignancies, with high rates of mortality and morbidity. The multifactorial nature of the disease requires appropriate risk stratification to enable the most appropriate preventive measures to be adapted and implemented according to the characteristics of the patient. In this sense, the present research aims to identify recent risk factors and environmental control measures against invasive aspergillosis to establish preventive actions to reduce the incidence of invasive aspergillosis in hospitals. (2) Methods: We conducted a qualitative systematic review of the scientific literature on environmental risk factors and preventive measures for invasive aspergillosis in patients with hematologic malignancies. The Medline, Cochrane, and Scopus databases were consulted, following the PRISMA and STROBE guidelines. (3) Results: Adequate implementation of environmental control measures is presented as the most efficient intervention in terms of prevention to decrease the incidence of invasive aspergillosis in hospitals. Neutropenia, fungal contamination, insufficient environmental control measures in hospital and home settings, length of hospital stay, and anemia, are identified as independent risk factors. We show that HEPA, LAF, and Plasmair^®^ systems are suitable methods to reduce the concentration of airborne fungal spores. Antifungal prophylaxis did not significantly influence IA reduction in our study. (4) Conclusions: Proper professional training and environmental control measures in hospitals are essential for the prevention of invasive aspergillosis. We should optimize risk stratification for patients with hematologic malignancies. Antifungal prophylaxis should be complementary to environmental control measures and should never be substituted for the latter. Studies should also be undertaken to evaluate the efficiency of environmental control measures against IA at patients’ homes.

## 1. Introduction

Invasive fungal infections (IFIs) are a growing global health problem. Several factors such as the use of fungicides in agriculture, the development of new immunosuppressive therapies, biological and cytotoxic drugs, the increase in the number of hematopoietic stem cell transplants (HSCT), and the increasing use of invasive biomedical devices such as intravascular catheters, have contributed to this situation [1]. Historically, systemic infection of *Candida* spp., with or without associated candidiasis, was the most common IFI in patients with hematologic malignancies (HM) in our latitudes. However, in recent decades, there has been a decrease caused by the current effectiveness of azole antifungal prophylaxis [2]. On the contrary, the incidence of IFI due to *Aspergillus* spp. and other filamentous fungi has increased [3].

*Aspergillus* spp. is a filamentous hyaline fungus that is widely distributed in nature. Its natural habitat is the soil, which enables it to be isolated from the soil and dust. More than 300 species of *Aspergillus* are known, of which only a small number cause opportunistic infections in humans. *Aspergillus* fumigatus is the species that causes infection most frequently in humans. This is due to its small spore size of 2–3 microns and its higher thermotolerance with a germination capacity between 37 °C and 40 °C. These characteristics optimize its pathogenicity [3,4]. However, there are other species of *Aspergillus* spp. that cause infection, such as *A. flavus*, *A. niger*, *A. terreus*, *A. nidulans*, and *A. lentulus*, which are becoming increasingly common, depending on geographical factors, host type, and antifungal prophylaxis used [3,5].

People with HM are the most susceptible group to suffer from IFI, both in the adult and pediatric population. Specifically, invasive aspergillosis (IA) is the most common invasive fungal infection in patients with acute hematologic malignancies [5]. Multiple risk factors are involved in the development of IA in patients with HM [6]. Many of them are related to the characteristics of the patients, such as: (i) neutrophils < 500 per mm^3^ and neutropenia time greater than 10 days [7], (ii) being older than 65 years [4,5], (iii) genetic polymorphisms associated with the immune response [5,6,7,8], (iv) type of neoplastic disease and its progression [9], (v) CD4 T lymphocytes < 200 per mm^3^ [3] and (vi) previous infections [4,6,10,11,12]. A history of repeated blood transfusions [13], the use of corticosteroids [14], intensive chemotherapy regimens with radiotherapy [15], and the use of new therapeutic strategies, such as biologic agents [16,17,18], increase the risk of IA in patients with HM.

Given the high morbidity and mortality of IA in patients with HM, good prevention strategies are essential to prevent this serious disease. Prevention measures can be stratified into four levels: (i) hospital environmental prevention, (ii) nosocomial infection control, (iii) prophylaxis, and (iv) out-of-hospital environmental prevention. Antifungal prophylaxis is strongly recommended for high-risk patients to prevent IA [19,20,21]. According to the latest consensus document developed by European societies, the main antifungal drugs recommended depending on the risk of the patient are: posaconazole, liposomal amphotericin B, itraconazole, and voriconazole [21,22].

Prevention of nosocomial infection is achieved thanks to the adequate training of the healthcare personnel responsible for high-risk IFI patients and therefore the use of scores, such as EQUAL 2018, which enables reporting on the quality of clinical care provided [22]. In addition, all of this is aided by the creation of IFI incidence records which facilitates the detection of increases in IFI incidence, to act accordingly in the event of outbreaks by performing cultures, verifying facilities and correcting deficiencies found [23].

Environmental risk factors are related to an increase in the number of conidia per cubic meter [1,2,3,4,5,24]. These can be produced naturally through seasonal changes or exposure to plant products, or artificially produced due to the proximity of construction areas, including in the hospital itself, or due to the lack of adequate insulation in the rooms of patients with HM, such as: (i) the inadequate insulation of doors and windows, (ii) a lack of High Efficiency Particulate Air (HEPA) filters, (iii) no laminar air flow (LAF), (iv) no positive differential pressure, and (v) a low number of air renewals.

High-risk patients with HM should be housed in hospital rooms with a protected environment, which are characterized by the presence of protected air. This protected air is achieved through the following: (i) the presence of HEPA filters, (ii) the implementation of mobile air decontamination systems, (iii) independent zones from the rest of the hospital, and (iv) construction materials that do not release particles [23,25].

Antifungal prophylaxis and infection control measures are the most widely implemented with wide consensus in the prevention of IA [26]. However, there is a lack of consensus on the level of airborne conidia that should be considered normal in different environments, which makes it difficult to establish clear guidelines for reducing exposure. Furthermore, the impact of environmental measures on the prevention of invasive aspergillosis is difficult to measure due to the complexity of the disease and the factors that contribute to its development [27]. 

The purpose of this research is to identify recent risk factors and environmental control measures that fight against the development of invasive fungal *Aspergillus* infection in patients with acute hematologic malignancies (AML) to establish preventive actions to reduce the incidence of invasive aspergillosis in hospitals.

## 2. Materials and Methods

### 2.1. PICO Question

Our research question was: “What environmental control measures are more effective in preventing the development of invasive fungal *Aspergillus* infections in hematologic patients?” This was transformed into the following PICO question:

- P (population): patients with acute hematologic neoplasms, (HN) acute myeloid leukemia (AML)/recipients of hematopoietic progenitor cell transplantation;
- I (intervention): environmental control measures;
- C (control): does not apply;
- O (outcome): invasive aspergillosis.

### 2.2. Study Selection

The review of the retrieved papers went through a five-stage process. Initially, articles were searched, followed by the elimination of duplicates. The next steps involved evaluating the titles and abstracts of the potentially relevant papers identified. Subsequently, the full texts of the selected articles were thoroughly examined, and their quality was evaluated. Throughout all stages, two independent groups of reviewers (D.R.P. and E.G.C.) and (J.R.P. and F.M.M.) conducted the review, with a third group of independent reviewers (J.C.C.V. and A.V.A.) involved in cases where there was disagreement.

### 2.3. Inclusion Criteria

1. Scientific articles from the years 2009–2023;
2. Articles published in Spanish and English;
3. Types of studies: Experimental studies, observational studies;
4. Articles containing the key descriptors: “Aspergillosis”, “Invasive pulmonary aspergillosis”, “Hematologic neoplasms”, “Hematopoietic Stem Cell Transplantation”, “Leukemia”, “Risk factors” and “Prevention”.

### 2.4. Exclusion Criteria

1. Articles that after applying the STROBE guidelines, for observational studies, respectively, did not reach a minimal punctuation;
2. Articles that do not address our research question;
3. Articles in pediatric populations.

### 2.5. Data Extraction

Data extraction was carried out by two reviewers who independently worked and followed the Center for Reviews and Dissemination and PRISMA guidelines [28,29]. Information from each included study was extracted and documented. The extracted data encompassed various aspects, including the year of publication, country, study design, quality assessment, sample size, target population, description of interventions, outcome measures, and study results.

### 2.6. Methodological Quality Assessment

The methodological quality was evaluated using the STROBE statement for observational studies [30]. All studies with 50% or less checked items were excluded for review. In all cases, the evaluation was conducted by two independent reviewers. 

### 2.7. Data Synthesis and Analysis

Data obtained from all studies that met inclusion criteria were organized and presented in tabular form. The tables include information about the study authors, sample characteristics, measurement of outcome variables, and key results. A qualitative synthesis was conducted, incorporating all identified studies, and the findings are presented in the tables.

## 3. Results and Discussion

**Scheme 1:**
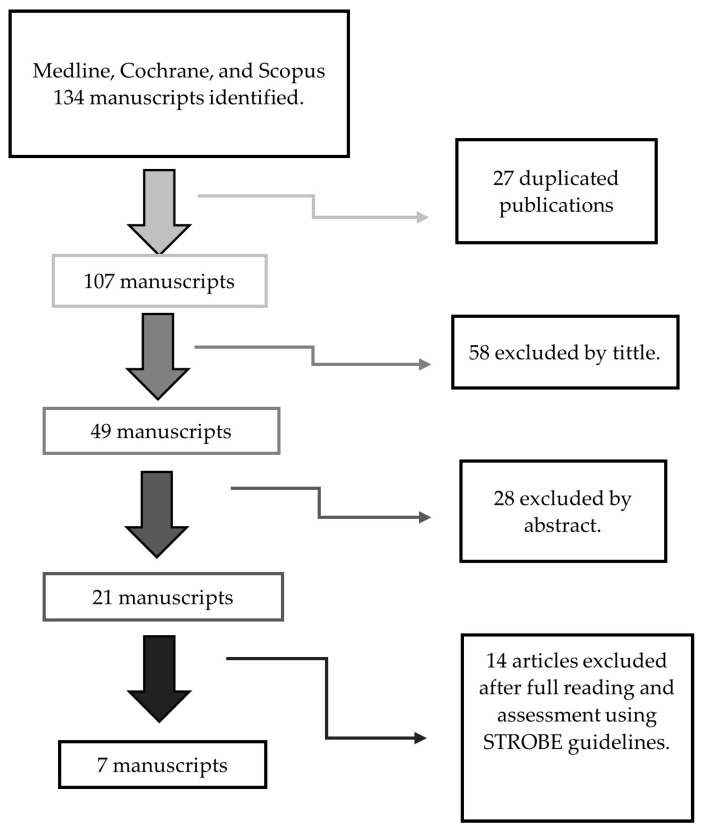
Diagram flow of the research selection strategy: first filter duplicated; second filter, title read; third filter, abstract read; fourth filter, full reading and STORBE guideline.

The research methodology went through a five-stage process. The article selection process is detailed in Scheme 1. We selected Medline, Cochrane, and Scopus databases for our search. The search strategy is detailed in Table 1. Initially, 134 articles were identified using the three databases. We eliminated 27 duplicate publications.

We obtained findings from seven studies in our research. Four of them are based on the environmental control measures established in hospitals during building construction [31,32,33,34]. High efficiency particulate air filtration (HEPA) and laminar air flow (LAW) are the main environmental control measures mentioned, presented in four out of seven search studies [31,32,34,35]. Research should be highlighted that studies a new mobile air decontamination system called Plasmair^®^ [36]. All are evaluated in hospitals [31,32,33,34,35,36,37] and one includes air analysis at home [37]. Two studies included antifungal prophylaxis as a preventive measure for IA [31,34]. Their main features are detailed in Table 2.

### 3.1. Risk Factors for IA

The risk factors for IA can be divided into two groups. Infection-related risk factors, including neutropenia, anemia, and the duration of hospitalization, and environmental-related risk factors, including fungal contamination and a lack of environmental control measures. The most common risk factor was neutropenia, followed by environmental factors, including fungal contamination and the absence of environmental control measures. Finally, other infection-related risk factors such as anemia and duration of hospitalization were also identified.

Neutropenia was evaluated as a risk factor in four of the seven studies of this investigation [31,33,35,37]. The incidence of IA was higher when the duration of neutropenia was longer than 7 days (Odds Ratio (OR): 9.95 (95% CI: 2.86–34.94) [31]. A third of patients who had neutropenia for more than 40 days developed IA [35]. IA occurred in 10 patients with severe neutropenia of the 14 patients with IA in the study by Rocchi et al. [37]. Neutropenia was strongly correlated with IA in the study by Loschi et al. [33]. These results confirm the overwhelming role of neutropenia in IA, as other previous studies and reviews showed [38,39,40,41]. The other is anemia, which has a positive correlation with IA in the study by Friese et al. (OR: 1.044 (95% CI: 1.008–1.081) [35]. We could highlight that, to our knowledge, this is the first time that this association is reported in the literature.

Other risk factors for IA in patients with HM are also mentioned in some of the studies in our investigation. The duration of hospitalization is one of them that was strongly correlated with IA in the study by Loschi et al. [33].

Fungal contamination less than 0.1 CFU/m^3^ in a HEPA filtered area or under 5 CDF/m^3^ in an isolation area is enough to trigger IA in hospital patients [42]. Two of the investigations in this review established the presence of fungal contamination as predictor variables for the development of IA [34,37]. In the study by Rocchi et al., 5 of 14 patients who developed IA were concomitant with abnormally high levels of *A. fumigatus* in the hematology corridors of the ICU (14–25 CFU/m^3^ [37]). In the same study, another 5 of the 14 IA patients, although there was no fungal contamination during hospitalization, were exposed to A. fumigatus and A. flavus at home (9–48% [37]). Therefore, there is a risk of developing IA in HM patients when there is a significant exposure to *A. fumigatus* and *A. flavus* in the hospital or even at home [37]. Park et al. demonstrated that the total incidence rate of IA was significantly higher during hospital demolition and excavation work than during construction, when airborne fungal contamination was lower (9.95 vs. 5.60 CFU/m^3^ for total mold spores and 2.35 vs. 1.70 CFU/m^3^ for *Aspergillus* spp.) [34]. Herein, differences in spore levels are more pronounced for total mold spores than for *Aspergillus* spp. Therefore, total levels of mold spores could be a predictor of ineffective air filtration and/or the existence of conditions that favor mold settling, as well as an indirect marker of air fungal contamination [34]. Previous studies confirm that patients with hematologic malignancies are at increased risk for IA caused by molds, *Aspergillus* spp. being the most common pathogen [43], as well as corroborate a significant relationship between environmental fungal contamination on the hematology wards and the incidence of IA [44].

Construction activity at hospitals has been reported to be an independent risk factor for invasive fungal disease in several studies [45,46,47,48,49]. Two of our studies highlighted the lack of environmental control measures and/or the lack of maintenance of these measures as a significant risk factor for IA in patients with HM during building construction or renovation at hospitals [31,32]. In the study by Combariza et al., the absence of environmental control measures is an independent risk factor for IA (OR: 2.99 (95% CI: 1.20–7.41) [31]. After long-term use of LAF systems in the hospital of Iwasaki et al.’s study, the risk of aspergillosis increased (Hazard Ratio (HR): 5.65). These results suggest that more efforts should be made to establish appropriate environmental control measures, as well as adequate maintenance protocols for filtration systems [32].

### 3.2. Environmental Preventive Measures against IA

**Figure 1:**
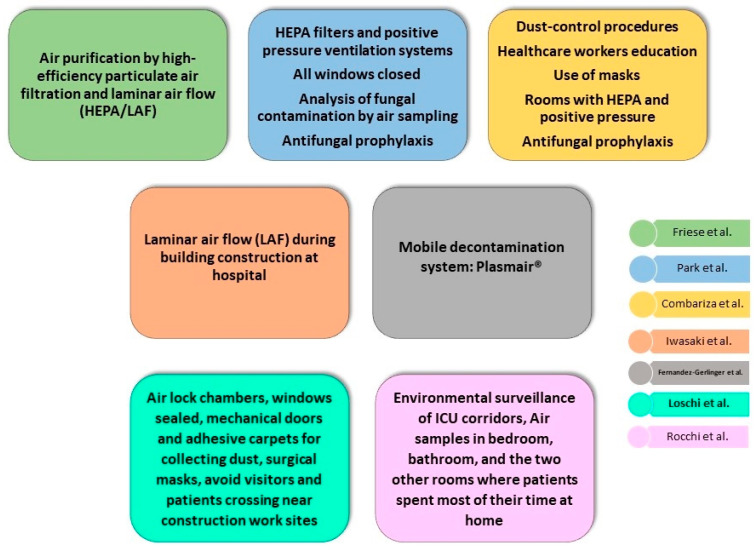
Environmental preventive measures against invasive aspergillosis in patients with hematologic malignancies [31,32,33,34,35,36].

All our studies implemented some kind of environmental control measures in their research that are detailed in Figure 1.

The absence of appropriate environmental prevention measures increased the incidence of invasive aspergillosis (IA) in patients with HM during hospital building renovation/construction [31,33]. Proper pre-planning before construction/renovation work is essential to establish optimal prevention guidelines based on the needs of an at-risk population. In this planning, the adequate training for healthcare personnel is highlighted. These measures must be more restricted during the phases of excavation/demolition [34].

Three studies [31,33,34] emphasized the effectiveness of physical barriers during hospital construction/remodeling for avoiding IA infection. With Combariza et al.’s environmental control measures (Table 2), the incidence of IA was reduced from 25.8% to 12.4%. These environmental control measures were protective for IA with a relative risk of 0.595 (95% CI: 0.39–0.90) [31]. The incidence of IA was higher in the ward without any ventilation system and with the highest total mold and *Aspergillus* spp. in the study by Park et al. (Table 2). Furthermore, they highlight that the incidence of IA was higher in the demolition and excavation period, when airborne fungal spore levels tended to be higher [34]. The environmental control measures established by Loschi et al. (Table 2) were sufficient enough to prevent invasive pulmonary aspergillosis in patients with neutropenia [33].

In hospital building renovations, air filtration is a proven protective factor against IA [33]. The Centers for Disease Control (CDC) and Prevention guidelines recommend HEPA filter systems for high-risk patients to reduce IA in hospitals during construction and renovation. In the study by Combariza et al., the most effective measure to prevent IA was the isolation of patients with HM in HEPA filtered rooms (RR: 0.98; 95% CI: 0.84–0.94) [31]. Fungal contamination was lower in the HEPA filter-equipped wards in the study by Park et al. [34], enabling the reduction of IA in these wards of the hospital. Another effective procedure to avoid aspergillosis is the isolation of the LAF. Iwasaki et al. demonstrated that the LAF system reduces the risk of aspergillosis during the construction of a new hospital (HR: 1.97), although it increased when installed on a new hematology ward (HR: 5.65) [32].

HEPA and LAF systems are suitable methods to reduce the concentration of airborne fungal spores. In a study carried out in patients without transplantation with HM, the implementation of HEPA/LAF was associated with a >50% risk of neutropenia-related IA [35]. IA was significantly less frequent under HEPA/LAF than under ambient conditions (OR: 0.097; 95% CI: 0.010–0.923). Its reduction was also observed in patients with a fatal hospitalization outcome (OR: 0.077). In 2010, in a large acute tertiary care hospital in Singapore, researchers demonstrated that portable HEPA filters were effective in the prevention of IA (OR: 0.49 (95% CI 0.28–0.85) [50]. A study carried out in immunosuppressed patients also demonstrated the efficient prevention measure of HEPA with or without LAF against IA [51]. Therefore, we can confirm that high-risk patients with HM should be isolated in hospital areas with HEPA/LAF systems to avoid IA. However, the availability of such systems in hospital areas is limited. For this, portable environmental decontamination equipment has emerged, enabling the administration of high-quality air according to the needs of high-risk patients with HM. Fernandez-Gerlinger et al. [36] demonstrated that a mobile air-decontaminated system called Plasmair^®^ reduced the incidence of IA in these patients (OR: 0.11; 95% CI: 0.00–0.84).

The Infectious Diseases Society of America (IDSA) recommended antifungal prophylaxis for the management of aspergillosis in 2016 [52]. However, this prophylaxis did not significantly influence IA incidence in our study. The combined administration of antifungal agents and properly applied environmental prevention measures are not better than the isolated application of environmental prevention measures in terms of reducing the risk of IA, as reported by Combariza et al. [31]. These results are also observed by Friese et al. [35]. Previous studies, such as one carried out in 2001, corroborate these results. Oren et al. [53] compared environmental control measures with antifungal prophylaxis against IA in acute leukemia patients, demonstrating that HEPA reached the absence of new cases of IA in patients hospitalized in rooms with this filtration system, while a partial reduction in IA incidence (from 50% to 43%) was achieved with antifungal prophylaxis. Moreover, ISDA recognized that although there is an optimal antifungal therapy against IA, the mortality rate of the disease remains high [52]. Therefore, although antifungal prophylaxis is a priority measure for high-risk patients, it could not be considered a substitute for the proper implementation of mechanical prevention measures.

### 3.3. Main Recommendations to Fight against IA

We also collected the main proposals given by the researchers based on their studies to combat IA in patients with HM. In order of importance, they are: environmental control measures, personalized risk stratification, a multidisciplinary committee, the monitoring of fungal contamination, and fungal prophylaxis.

The priority for most of the studies reviewed (6 of 7) was to establish appropriate environmental control measures against IA [31,32,33,35,36,37]. Combariza et al. concluded their study with the effectiveness of the implementation of environmental control measures during hospital construction activity to prevent IA in acute leukemia patients [31], as did Loschi et al. [33]. Rocchi et al. supports that these preventive measures should also be achieved in patients´ homes. For this, they suggested giving advice such as avoiding activity in highly contaminated rooms (a garage, for example), cleaning all rooms, or even installing air treatment devices [37]. However, previous studies found difficulties controlling the environment of the patient at home [51], suggesting more studies in this sense are needed to address this problem.

The HEPA, LAF, and Plasmair^®^ systems are well established as efficient protective factors against IA [32,35,36]. As these systems are quite expensive and not available to all patients, all researchers agreed to leave this service to the most compromised patients, which could be immunocompromised patients [32] with episodes of neutropenia for 10 days or more [35]. The European Society for Clinical Microbiology and Infection Diseases (ESCMID) gave some recommendations in 2016 on how to deal with IA in patients with HM, suggesting that these patients should be segregated into subgroups and provided a specific classification for each of them, including environmental measures in prevention [21]. Loschi et al. suggest that a multidisciplinary committee should make the decision about who could be the beneficiary of the different mechanical preventive measures, as the CDC recommends [33]. The risk stratification for IFI made by Pagano et al. [38] in their review should be highlighted, offering it as a useful tool for optimizing diagnostic procedures and therapeutic strategies for preventing and treating IFI in patients with hematologic malignancies. 

Monitoring airborne fungal spore levels is recommended during construction periods in hospitals with immunosuppressed patients by Park et al. [34], as well as by Rocchi et al. [37]. These last researchers also recommend the assessment on patients´ homes, with an environmental survey and monitoring levels, focusing on A. fumigatus and *A. flavus* [37]. Previous studies also underline the importance of environmental surveillance for the application of strict preventive measures against IA [44]. Iwasaki et al. also add fungal prophylaxis in patients who are highly exposed to environmental factors [32]. Diaz-Arevalo and Kalkum suggest new antifungal vaccines as future prophylaxis treatment, reducing sensitivity to IA in high-risk patients [54]. Ruiz Camps [15] point out, in an editorial letter, that there are no studies that address the definition of the optimal duration of antifungal therapy, which inspires us to think about how to handle it without further ado, when we will need to decide which patients at risk should receive prophylaxis, for how long and with what drug. Other recommendations included a delay in hospital discharge when necessary, as well as the introduction of health monitoring before patients return home if there is a suspicion of high-risk IA [37].

### 3.4. Strengths and Weakness

This research synthesizes and points out the main risk factors related to IA in patients with HM mentioned in the recent scientific literature. We also highlight the environmental preventive measures that several researchers have considered in hospitals, in building construction, and in the homes of patients with HM fighting against IA. In addition, we emphasize the main recommendations made to combat IA in these patients around the world.

Publication and selection biases are the main limitations of this study. We established clear inclusion and exclusion criteria to reduce these biases with the greatest possible objectivity, to prevent the results from being distorted. Language and the year of publication could be another bias, as we only included studies from the last 10 years (2013–2023) written in Spanish or English. Other aspects that could compromise the validity of the results obtained in this research are the quality of the original studies included, the variability between studies, or errors in the analysis phase. The conclusions of the research are largely dependent on these aspects. Two of us analyzed each study with the Prisma and Strobe guidelines [29,30] to minimize this last bias.

Additionally, the studies differ in sample sizes, study populations, and methodologies, introducing potential variations in reported incidence rates. Certain studies have limitations, such as being conducted in a single center or not measuring specific variables, which can influence the accuracy and generalizability of the findings. Therefore, it is important to compare the incidence rates of IA with caution in these studies.

## 4. Conclusions

We identified recent risk factors for IA associated with IA patients. Neutropenia is the main risk factor, followed by fungal contamination in hospitals and patients’ homes, and lack of proper environmental control measures. The duration of hospitalization and anemia are also mentioned as risk factors in our research. We also demonstrated the effectiveness of physical barriers during hospital construction/remodeling for avoiding IA in patients with HM. HEPA, LAF, and Plasmair^®^ systems are suitable methods to reduce the concentration of airborne fungal spores. Antifungal prophylaxis did not significantly influence the reduction of IA in our study. Although IDSA recommends it, we suggest not considering this prophylaxis as a substitute for physical barriers, but as an additional treatment for high-risk patients. Establishing proper environmental control measures against IA is the key priority recommendation in most of the studies researched. These mechanical preventive measures should go along with the professional education of healthcare workers and multidisciplinary committees that adapt these measures according to the risk stratification of patients. Antifungal prophylaxis should be complementary to environmental control measures and should never be substituted for these latter factors. Environmental surveillance is also recommended not only in hospitals, but even in homes, where fungal contamination is a risk factor for IA. Studies should be undertaken to evaluate the efficiency of environmental control measures against IA in patients’ homes.

## Figures and Tables

**Table 1:** Source of data, search strategy, and number of manuscripts.

Source of Data	Search Strategy	Number of Manuscripts
PubMed	MeSH: (“Aspergillosis”[Mesh] OR “Invasive Pulmonary Aspergillosis”[Mesh]) AND (“Hematologic Neoplasms”[Mesh] OR “Hematopoietic Stem Cell Transplantation”[Mesh] OR “Leukemia” [Mesh]) AND (“Risk Factors”[Mesh] OR “Primary Prevention”[Mesh])	79
SCOPUS	(Aspergillosis OR “invasive pulmonary aspergillosis”) AND (“hematologic stem cell transplantation” OR “hematologic neoplasms” OR leukemia) AND (“Risk factors” OR “Primary prevention”) AND NOT index (MEDLINE).	46
Cochrane Library	“aspergillosis”	9

**Table 2:** Main results of the studies.

Authors/Year/Country ^1^	SD	SDu	Location	Patients	Sample	IAI	Assessment
Friese et al. [34]/2023/Germany	RCS	7	Hospitalization during construction of a new building with HEPA/LAF	AML or ALL	204 No HEPA/LAF 126 with HEPA/LAF	51 No HEPA/LAF 26 With HEPA/LAF	Efficiency of HEPA/LAF against IA
Park et al. [33]/2019/South Korea	PCS	0.5	Hospitalization during construction of new building with HEPA	Patients with HM	29	15 First period 14 s period	Environmental spore surveillance Preventive effect of HEPA system against IA
Iwasaki et al. [31]/2019/Japan	RCS	20	Hospitalization during construction of a new building with LAF	AML or ALL	124	14	Preventive effect of long-term LAF isolation against IA
Combariza et al. [30]/2017/Colombia	RCS	2.5	Hospitalization during construction of a new building with and without ECM	AML or ALL	175 62 No ECM 113 W ECM	29 16 No ECM 13 W ECM	Impact of ECM for the prevention of IA
Fernandez-Gerlinger et al. [35]/2016/France	RCS	2	HICU with and without Plasmair^®^	Patients with HM and CIN	156	11	Impact of Plasmair^®^ on IAI
Loschi et al. [32]/2015/France	PCS	5	HICU during hospital renovation	with neutropenia for more than 7 days	438	84	Effectiveness of mechanical preventive measures against IA
Rocchi et al. [36]/2014/France	PCS	2	HICU and PH	Hospitalized in the HICU	53	14	ES of HICU and PH

^1^ The studies are organized by year of publication. ALL = Acute Lymphoblastic Leukemia, AML = Acute Myeloid Leukemia, CIN = Chemotherapy-Induced Neutropenia, ECM: Ennvironmental Control Measures, ES = Environmental Surveillance, HEPA = High Efficiency Particulate Air, HICU = Hematology Intensive Care Units, HM = Hematologic malignancies, IA = Invasive Aspergillosis, IAI = Incidence of IA, LAF = Laminar air Flow, PCS = Prospective Cohort Study, PH = Patients’ homes, RCS = Retrospective Cohort Study, SD = Study Design, SDu = Study Duration (in years).

## Data Availability

Data sharing is not applicable to this article.
